# Evaluating the antimicrobial, apoptotic, and cancer cell gene delivery properties of protein-capped gold nanoparticles synthesized from the edible mycorrhizal fungus *Tricholoma crassum*

**DOI:** 10.1186/s11671-018-2561-y

**Published:** 2018-05-16

**Authors:** Arpita Basu, Sarmishtha Ray, Supriyo Chowdhury, Arnab Sarkar, Deba Prasad Mandal, Shamee Bhattacharjee, Surekha Kundu

**Affiliations:** 1 Molecular and Applied Mycology and Plant Pathology Laboratory, Department of Botany, University of Calcutta, 35, Ballygunge Circular Road, Kolkata, 700019 India; 2 Department of Zoology, West Bengal State University, Barasat, North 24 Parganas, Kolkata, 700126 India

**Keywords:** Green synthesis, Protein-capped gold nanoparticles, Antimicrobial, Apoptosis, Cancer cell gene delivery

## Abstract

Biosynthesis of gold nanoparticles of distinct geometric shapes with highly functional protein coats without additional capping steps is rarely reported. This study describes green synthesis of protein-coated gold nanoparticles for the first time from the edible, mycorrhizal fungus *Tricholoma crassum* (Berk.) Sacc*.* The nanoparticles were of the size range 5–25 nm and of different shapes. Spectroscopic analysis showed red shift of the absorption maxima with longer reaction period during production and blue shift with increase in pH. These were characterized with spectroscopy, SEM, TEM, AFM, XRD, and DLS. The particle size could be altered by changing synthesis parameters. These had potent antimicrobial activity against bacteria, fungi, and multi-drug-resistant pathogenic bacteria. These also had inhibitory effect on the growth kinetics of bacteria and germination of fungal spores. These showed apoptotic properties on eukaryotic cells when tested with comet assays. Moreover, the particles are capped with a natural 40 kDa protein which was utilized as attachment sites for genes to be delivered into sarcoma cancer cells. The present work also attempted at optimizing safe dosage of these nanoparticles using hemolysis assays, for application in therapy. Large-scale production of the nanoparticles in fermentors and other possible applications of the particles have been discussed.

**Electronic supplementary material:**

The online version of this article (10.1186/s11671-018-2561-y) contains supplementary material, which is available to authorized users.

## Background

With the unavoidable and wide-spread application of nanoparticles in agriculture, medicine and household products, there is increased pressure to understand the harmful effects and ways to reduce it [1]. The green synthesis of nanomaterials using live organisms or their enzymes has the advantage of being ecofriendly, cost-effective, and safe for therapeutic uses [2]. Among microbes, filamentous fungi have a greater capacity to synthesize nanoparticles due to their ability to secrete greater quantities of enzymes [3, 4]. Extracellular gold nanoparticles (AuNPs) have been produced using several fungi [5–7], but only a few mention of natural protein coats over these particles in these reports [8, 9].

In medicine, with the arrival of multidrug resistant bacteria, nanoparticles are the alternate choice since these do not give rise to resistance [10]. AuNPs specifically hold promise in therapy and diagnostics of tumor cells and nanocarrier-based gene delivery [11–13]. Under physiologic conditions, AuNPs show low permeability through cell-membrane, but in tumor cells, the uptake is enhanced due to enhanced permeation and retention (EPR) effect [14]. This uptake is further enhanced when the AuNPs are capped with proteins. The protein cap helps to stabilize the nanoparticles in the colloidal state and provide docking sites for drugs or genes for delivery [14]. However, the process of capping involves additional steps. The provision of natural protein cap eliminates the application of chemical routes which are mostly hazardous [5]. In spite of its importance, there are limited reports involving green synthesis of noble metal nanoparticles with natural protein coats [15, 16].

Before application of AuNPs in therapy, an important aspect to be assayed is the biocompatibility of a nanomaterial with the cellular membranes [6]. Another aspect is cytotoxicity and AuNPs can aggregate red blood cells [13]. Therefore, emphasis needs to be placed on the hazardous aspects and use it within safe limits [7, 17].

Our laboratory reported thus far the only edible mycorrhizal fungi to produce extra-cellular silver nanoparticles, the fungus being *Tricholoma crassum* (Berk.) Sacc [2]. Here, we describe the green synthesis of AuNPs from *T. crassum* of the size range 5–25 nm and of different shapes. These were characterized and assayed for antimicrobial activity against bacteria, fungi as well as multi-drug-resistant (MDR) pathogenic bacteria. These had inhibitory effect on the growth kinetics of bacteria and potency of fungal spores. Most importantly, the particles are naturally protein-coated. We have tested these particles for their efficacy as a vehicle for gene delivery into cancer cells. Hemolysis assay was done to check the biocompatibility and toxicity of these particles. The apoptotic properties of the AuNPs were tested with comet assays on eukaryotic cells to estimate a workable concentration for therapeutic use with minimal side effects. The present work thus attempts at optimizing synthesis and application of the AuNPs at the medical and nanotechnological interface within safe limits so as to cause minimal environmental and biological damage.

## Methods

### Fungi, bacteria, and plant growth conditions

*Tricholoma crassum* (Berk.) Sacc. was used for nanoparticles production. For antimicrobial assays, *E. coli* (DH5α), *Agrobacterium tumefaciens* (LBA4404), multi-drug-resistant (MDR) strains of *E. coli* (DH5α), and *A. tumefaciens* (LBA4404) were used. The plant pathogenic fungi *Magnaporthe oryzae* and *Alternaria solani* were used. Tomato (variety Pusa Ruby) and tobacco (Variety SR1) seedlings were grown on soil in growth chambers with 16:8 h light/dark photoperiods, 28 ± 1 °C and light intensity of 50 μmol m^− 2^ s^− 1^.

### Synthesis of AuNPs

*T. crassum* mycelium was cultured in potato dextrose broth (PDB) for 7 days at 28 °C. 1 g of mycelial mat was agitated with 10 ml of deionized water on a shaker at 50 RPM for 24, 48, and 72 h at 28 °C. The supernatants were filtered through Whatman filter paper no. 1. The cell filtrate (pH 5.2) was incubated with 1 mM aqueous solution of chloroaurate (HAuCl_4_) and agitated at 28 °C in dark according to our report [2] for 1 h for each type of cell filtrate made by 24, 48, and 72 h of incubation with mycelium.

For biosynthesis of AuNPs at different pH, 1 M HCl or 1 M NaOH was used to adjust the pH of the cell-free filtrate to acidic range (3.5) and alkaline range (7, 8, and 9) prior to incubation. AuNPs were also synthesized using different reaction temperature (0, 15, 28, 75, and 100 °C), different concentration of cell-free filtrate (× 0.5, × 1, × 2) and chloroaurate ions (0.5, 1, 2 mM).

### UV-visible spectroscopy

Absorbance of supernatants from each cell filtrate incubated for 1 h with chloroaurate solution was analyzed using UV-visible spectrophotometer between 450 and 750 nm was used to plot the absorbance spectra.

### Scanning electron microscopy (SEM), transmission electron microscopy (TEM), atomic force microscopy (AFM), and X-ray diffraction (XRD)

These analyses were done according to Chowdhury et al. [15] with few modifications. Gold nanoparticles produced using 24 h cell filtrates followed by 1 h of incubation with 1 mM HAuCl_4_ solution at 28 °C (pH 5.5) was characterized using scanning electron microscopy (SEM), transmission electron microscopy (TEM), atomic force microscopy (AFM), and X-ray diffraction (XRD). A thin film of the AuNPs on a glass stub was vacuum dried and was subjected to SEM using FEI Quanta 200 (FEI, USA).

The shapes and sizes of the AuNP were determined by TEM. A drop (10 μl) of the AuNP suspension was placed on carbon-coated copper grids and was subjected to vacuum desiccation before loading onto a specimen holder. TEM micrographs of these nanoparticles were obtained using TECNAI G TEM with a low voltage (100 kV) construction.

For AFM imaging, AuNPs were deposited onto a freshly cleaved muscovite Ruby mica sheet (Ruby Mica Co. Ltd., India) and were dried by using a vacuum dryer. Acoustic alternative current (AAC) mode AFM was performed using a Pico plus 5500 ILM AFM (Agilent Technologies, USA).

For XRD study, a thin film of AuNP suspension was spread evenly on a glass slide and was dried by using vacuum dryer. XRD patterns were recorded in a D8 Advance DAVINCI XRD System (Bruker AXS Pvt. Ltd.) operated at a voltage of 40 kV and a current of 40 mA with CuKα radiation (λ = 1.54060/1.54443 Å), and the diffracted intensities were recorded from 35° to 80° 2θ angles.

### Computer software analysis

The measurements of AuNPs and construction of a histogram was done using OLYMPUS software MEASURE IT tool. The concentration of nanoparticles was calculated according to Sriram et al. [18] and our previous publication, Chowdhury et al. [15].

### Transformation of bacteria to develop multi-drug resistance

*A. tumefaciens* strain LBA4404 and *E. coli* strain DH5α were made multi-drug resistant by transformation using the plasmids pCAMBIA2301 and pUC19 with pZPY112, respectively, using our published protocol [2, 15].

### Antibacterial assays and bacterial growth assays

These assays were done according to our published protocol [15]. Gold nanoparticles, that were synthesized using 24 h cell filtrates and 1 h of incubation with 1 mM HAuCl_4_ solution at 28 °C (pH 5.5), were used for all biological assays. For paper disc assays, increasing amounts of polydisperse AuNPs (0.249, 0.498, 0.747, 0.996, 1.245 μg) were used. From fresh overnight cultures of each bacterial strain, 25 μL aliquot was spread on to LB agar plates. Dilution series of the nanoparticle solution were made up using AuNP solution of concentration 31.121 mg/L and sterile deionized water. Sterile paper discs of 5 mm diameter with increasing amount of gold nanoparticles in each disc such as 0.249, 0.498, 0.747, 0.996, and 1.245 μg (in a total volume of 40 μl) were placed on the bacterial plates and incubated. *A. tumefaciens* plates were incubated in 28 °C for 48 h and *E. coli* at 37 °C for 12 h. For growth assays of DH5α and LBA4404, 7.5 ml bacterial culture was supplemented with 2.5 mL of AuNPs.

### Antifungal assay

Aqueous suspension of *M. oryzae* spores was made at 6.1 × 10^5^ spores/ml with a hemocytometer. 150 μl of this suspension was spread on MEA plate. Sterile paper discs of 5 mm diameter with increasing amounts of polydisperse AuNPs (0.249, 0.498, 0.747, 0.996, and 1.245 μg) were placed on the plates and incubated at 28 °C. Inhibition zones were measured after 2 days.

### Antimicrobial assay for bacterial cells treated with AuNPs

Over-night liquid LBA4404 culture was treated with equal volume of AuNP suspension (15.56 mg/L) for 12 h in 28 °C. 50 μl of the suspension was mixed with equal volume of 0.4% Trypan blue solution (0.5 g trypan blue, 500 ml glycerol, 450 ml distilled H_2_O, 50 ml HCL) was observed under compound microscope (Leica DMLS, Germany).

### Fungal spore germination assay in presence of AuNPs

A dilution series of nanoparticles was made with 20, 40, 60, 80, and 100% *v*/*v* using stock solution of nanoparticles of concentration 15.56 mg/L making the final volume to 100 μl with water. Equal volumes of these suspensions were added to 50 μl *Alternaria solani* spore suspension (4.2 × 10^5^ spores/ml) and incubated at 28 °C. The spores were observed at 0, 2, 4, and 6 h under compound microscope (Leica DMLS, Germany).

### Comet assays

The apoptogenic properties of the AuNPs were measured by standard comet-assay [19] with few modifications. The tobacco or tomato leaves were exposed to increasing concentrations (0, 15, 20, and 30% *v*/*v* for tobacco; 5, 10, 15, and 20% *v/v* for tomato) of AuNPs (15.56 mg/L stock) for 24 h. Electrophoresis of isolated nuclei was conducted at 0.74 V/cm (25 V, 300 mA) for 30 min at 4 °C. The slides were neutralized with 0.4 M Tris buffer, dehydrated in methanol, stained with ethidium bromide (20 μg/ml), and observed under a fluorescence microscope with excitation filter of 515–560 nm and barrier filter of 590 nm. The data was analyzed with Tritek CometScore software.

### SDS-PAGE

Proteins were isolated according to our publication [15]. For analysis of proteins bound to the nanoparticles, the AuNPs were washed with sterile water and boiled in Laemmli buffer for 10 min and centrifuged at 8000 rpm for 10 min. The SDS-treated and untreated samples were run on 12% SDS-PAGE.

### Cancer cell line culture

Sarcoma 180 cell line was cultured in RPMI 1640 medium [20] containing 10% fetal bovine serum, 200 U/ml penicillin, and 200 μg/ml streptomycin at 37 °C, 5% CO_2_ in humidified incubator.

### Delivery of plasmid DNA-AuNP complex into cancer cells

Plasmid was isolated from DH5α containing pCAMBIA1302 using published protocol [15]. The plasmid containing the construct of *gfp* sequence cloned under pCaMV35s promoter was delivered into Sarcoma 180 cells using standard protocol [21]. The cells were viewed under fluorescence microscope using filter specific for GFP (excitation maximum = 395 nm) (Axioskop-40, Carl Zeiss). Cells treated with naked plasmid DNA was kept as a control.

### Hemolytic assay

Hemolytic assay was done following standard protocol [22]. Equal volumes of erythrocytes (1.6 × 10^9^ erythrocytes/mL) were treated with varying concentrations of AuNPs (0.1, 0.5, 1, 5, 10, and 20 μl/mL from a stock 15.56 mg/L) for 1 h and hemolytic activities of the nanoparticles at different concentrations were calculated.

## Results and discussion

### Biosynthesis of AuNPs at pH 5.5

**Fig. 1:**
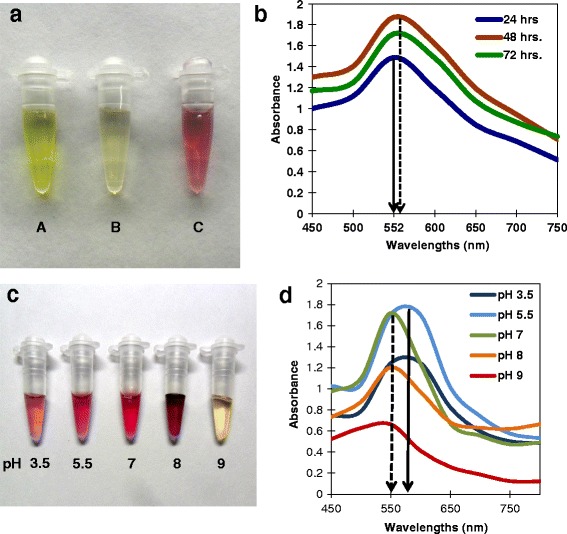
Biosynthesis of AuNPs using *Tricholoma crassum* and spectroscopic analysis. **a** Color change during reaction. **A** 20 mM solution of HAuCl_4_. **B** Mycelia-free cell filtrate of *Tricholoma crassum*. **C** 1 mM HAuCl_4_ with 24 h cell filtrate for 1 h showing violet color indicating synthesis of AuNPs. **b** UV-vis spectra of the AuNPs synthesized with different incubation periods (24, 48, 72 h). Solid arrow shows absorption peak at 552 nm for 24 h incubation period. Dashed arrow shows slight red shift of the absorption maxima with increase in incubation period. **c** AuNPs synthesized under different pH showing different colors. **d** UV-vis spectra of the same. Solid arrow shows absorption peak at 552 nm for pH 5.5 and dashed arrow indicates blue shift of absorption maxima with increase in pH

During synthesis, the formation of the AuNP is indicated by the change in color of the cell-filtrate from light yellow to violet [23] due to the change in surface plasmon resonance (SPR). 1 mM HAuCl_4_ solution without fungal filtrate was greenish yellow (Fig. 1a, ‘A’) and the cell-filtrate of *T. crassum* which was pale yellow (Fig. 1a, ‘B’). After incubation, the color of the mixture changed to violet within 1 h (Fig. 1a, ‘C’), indicating the formation of AuNPs.

One drawback of other methods of production of nanoparticles from microorganisms is that these are time-consuming and the organisms produce toxins. Previously, *Phanerochate chrysosporium* cell-free extract was utilized to produce AuNPs in 90 min [3]. Recently, silver and gold nanoparticles were biosynthesized using *Sporosarcina koreensis* using an extended 1–2 days reaction time [24]. In our report, the production time of AuNP has been reduced to 1 h only.

### Ultra-violet to visible spectroscopy

The optical absorption spectra of metal nanoparticles are governed by shape, aggregation, and the SPR, which shift in accordance with the particle size and shape [25, 26]. Figure 1b shows the UV-vis spectra of AuNPs synthesized using cell filtrates produced over 24, 48, and 72 h followed by 1 h of incubation with 1 mM HAuCl_4_ solution (pH 5.5). Here, the absorbance peak is at 552 nm for the 24 h cell filtrate. The transverse plasmon resonance band that appears first at 552 nm shifts slightly towards red from 24 to 48 and 72 h (shown as solid vs. dashed line Fig. 1b), confirming a red shift with progressively amplified intensity of absorbance. The broadening of the peaks indicates that the particles are polydispersed. The strong SPR centered at ca. 550–560 nm is typical of colloidal gold (Fig. 1b). As the concentration of the cell filtrate increased with increase in incubation periods (i.e., 24, 48, 72 h), the absorbance also increased proportionately. When the reaction was done at 28 °C, it reached equilibrium after 1 h and was stable over 30 days with no evidence of aggregation probably due to the stabilizing protein coat. In previous studies, BSA coated AuNPs showed no aggregation [27]. Further studies were done using AuNPs prepared with 24 h cell filtrate.

### Biosynthesis of AuNPs at different pH and UV-vis spectroscopy

The AuNPs were synthesized at different pH of 3.5, 5.5, 7, 8, and 9 resulting in pink to deep violet coloration (Fig. 1c). The UV-vis spectroscopy showed the absorbance maxima, and the SPR wavelength increased from pH 3.5 to pH 5.5 resulting in a red shift. However, with further increase in pH from 7 to pH 9, the absorbance maxima and the SPR wavelength decreased, showing a blue shift (Fig. 1d). The peak at pH 9 had a smaller amplitude and unchanged color, indicating only small amounts of AuNPs were formed. This being an enzymatic biosynthesis, pH 9 probably inhibited the enzyme reaction required for the formation of AuNPs (Fig. 1d). The nanoparticles produced at different pH did not aggregate after 1 month at room temperature.

### Production of AuNPs using different temperatures, substrate concentrations, and precursor concentrations

**Fig. 2:**
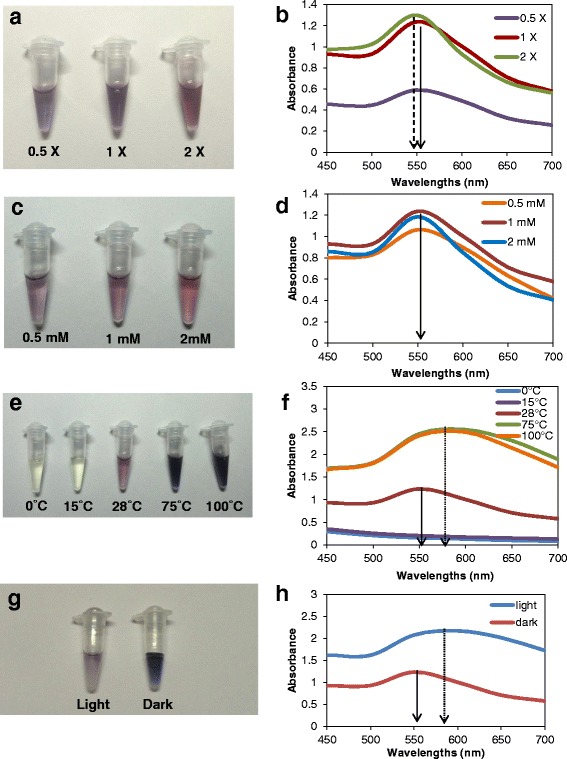
Biosynthesis and UV-vis spectroscopy of AuNPs from *Tricholoma crassum* using different synthesis parameters. **a**, **b** Different concentrations of cell-filtrate. **c**, **d** Different concentrations of HAuCl_4_. **e**, **f** Different reaction temperatures. **g**, **h** In dark and under light. Solid arrow shows the typical absorption peak at 552 nm for × 1 cell filtrate and 1 mM HAuCl_4_ at 28 °C pH 5.5 and dashed arrow indicates blue shift of absorption maxima, dotted arrow shows red shift in SPR band

Optimization of physico-chemical conditions during biosynthesis is critical for generation of functionally efficient nanoparticles [28]. Synthesis using higher concentrations of filtrate showed increased production of AuNPs with more color intensity and absorbance maxima (Fig. 2a, b). Slight blue shift of the maximum localized surface plasmon resonance (LSPR) was observed for synthesis with × 2 filtrate indicating increased inter-particle distance and decreased cluster size [29, 30].

**Fig. 3:**
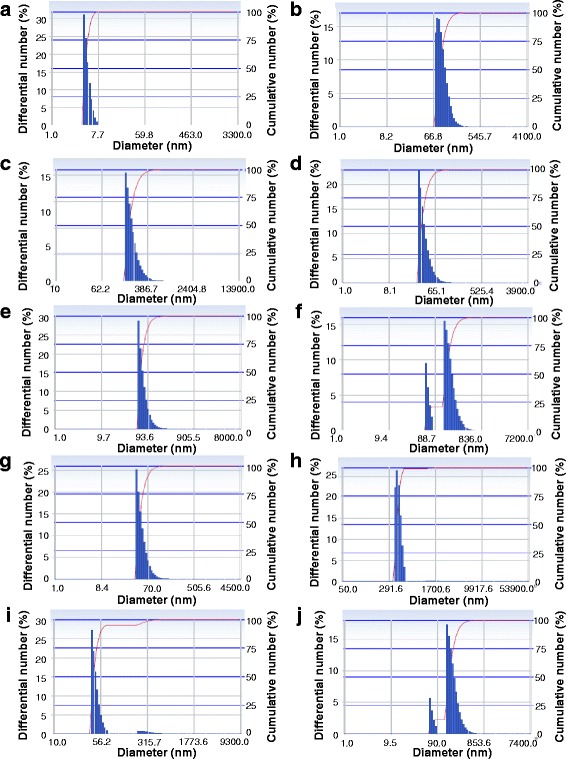
DLS showing size distributions of the synthesized AuNPs **a** with × 1 cell-free filtrate, 1 mM HAuCl4 at 28 °C in dark, **b** under light, **c** with × 0.5 cell-free filtrate, **d** with × 2 cell-free filtrate, **e** at 0 °C, **f** at 15 °C, **g** at 75 °C, **h** at 100 °C, **i** with 0.5 mM HAuCl_4_ and **j** with 2 mM HAuCl_4_

Of the three concentrations of gold chloride tested (0.5, 1, and 2 mM), synthesis was maximum for 1 mM, with absorption maxima at 552 nm. (Fig. 2c, d). 28 °C was found to be optimum for synthesis. Higher temperatures (75 and 100 °C) although mediated faster synthesis of AuNPs, the dark color and the redshift of the absorption maximum (Fig. 2e, f) indicated larger particles than at 28 °C. At 0 and 15 °C, there were no color development or absorbance peak within 550–600 nm. Synthesis in light resulted in deep violet coloration (Fig. 2g) and a redshift of absorption maximum with a broad peak (Fig. 2h) signifying larger particles. The particle size and number was assayed with DLS (Fig. 3a–j). This variation in the size and aggregation of nanoparticles depend on the nature and amount of associated organic matter [30] which again varies with the conditions of synthesis.

### Scanning electron microscopy (SEM) and transmission electron microscopy (TEM)

**Fig. 4:**
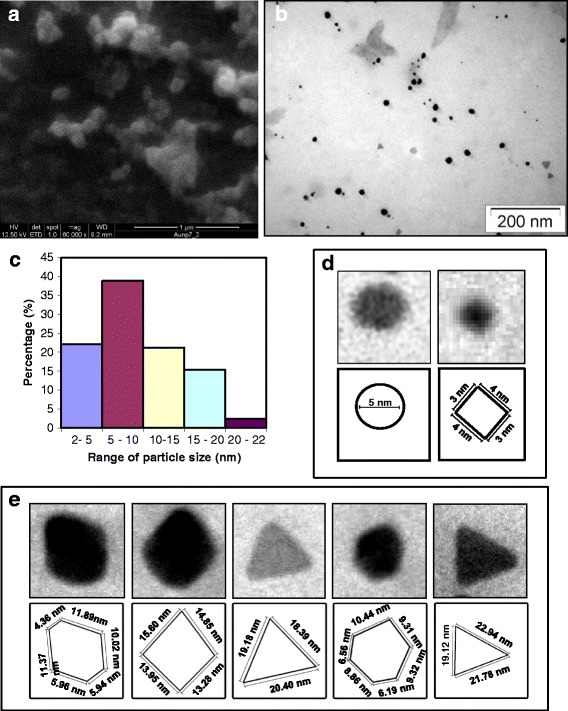
Electron microscopy of the AuNPs. **a** SEM at magnification of × 80,000. **b** TEM showing dispersed particles of different sizes in a microscopic field. **c** A histogram showing particle size distribution of the AuNPs. Enlarged view of individual AuNPs showing different geometric shapes and their diagrammatic representations with dimensions. **d** Spherical (left) and rhomboidal (right) of small size range. **e** From left: hexagonal, equilateral triangular, rhomboidal, polyhedral, and isosceles triangular

SEM showed AuNPs of small sizes and distinct geometric shapes at × 80000 magnification (Fig. 4a). TEM analysis clearly showed polydispersed AuNPs of the size range 2–22 nm diameter (Fig. 4b). The size distribution graph shows AuNPs of the size range 5–10 nm were of the highest frequency followed successively by 2–5 nm, 10–15 nm, 15–20 nm, and 20–22 nm (Fig. 4c). These were of different geometric shapes like small circular or rhomboid with diameter 5 nm or less, hexagons, cuboids, isosceles triangles, and near-equilateral triangles with sides varying from 4.36 to 22.94 nm (Fig. 4d, e).

### Atomic force microscopy (AFM)

**Fig. 5:**
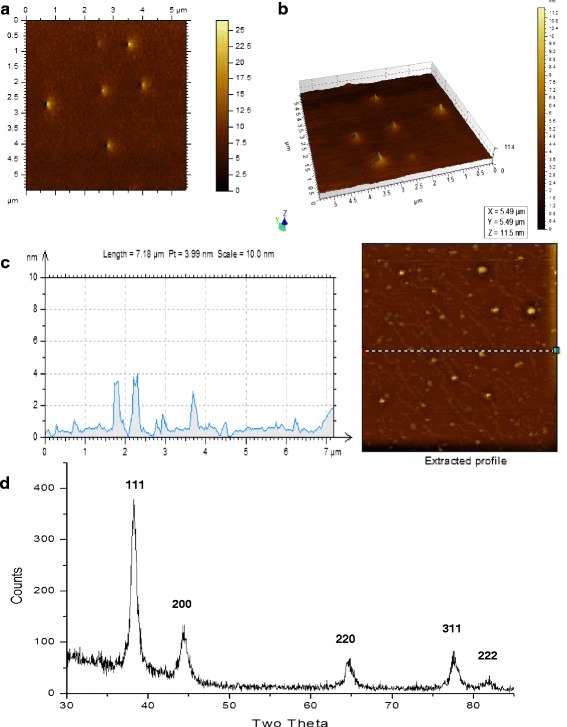
AFM and X-ray diffraction of the AuNPs. **a** AFM image: top view. **b** AFM image: three dimensional view. **c** An AFM image of AuNPs and graphical profile for heights of the nanoparticles lying on the dotted line in the field. **d** X-ray diffraction pattern of the nanoparticles showing peaks typical for gold

Figure 5a shows an AFM image of the dispersed AuNPs. The two-dimensional view (Fig. 5a) shows that the particles had nearly similar surface thickness. The heights of these particles were visualized with single surface 3D AFM (Fig. 5b). The AFM 2D graph of the AuNPs lying on a random linear zone (marked with dotted line, Fig. 5c) shows heights ranging from 1 to 4 nm. The plane surface plasmon band indicated that the thickness of the particles was smaller than the edge length.

### X-ray diffraction studies(XRD)

The XRD pattern of thin film of the particles revealed the presence of only AuNPs. A strong diffraction peak at around 38° is generally ascribed to {111} facet of the face centered cubic (FCC) structure [3]. The XRD pattern here showed a dominant diffraction peak at 38.23 attributed to the FCC structure. The diffraction peaks of other four facets were weaker. The four distinct Bragg diffraction peaks at 38.23, 44.31, 64.60, 77.58, and 81.63 closely matched that of AuNPs (Fig. 5d, Additional file 1: Table S1).

### Calculation of the concentration of AuNPs

The concentration of the nanoparticles [15] was found to be 15.56 mg/L for particles produced with 24 h cell filtrate incubated with 1 mM HAuCl_4_.

### Antimicrobial assay of the AuNPs using pathogenic bacteria and fungi

**Fig. 6:**
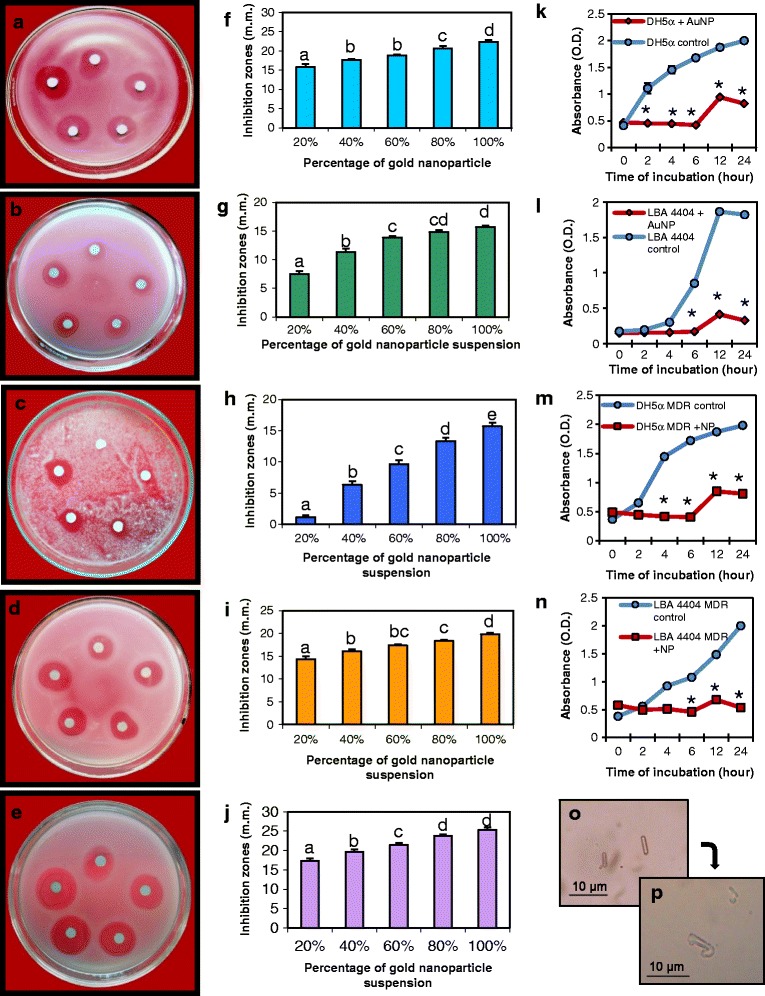
Assay of antimicrobial properties of AuNPs on pathogenic bacteria, fungi, and multi-drug-resistant (MDR) bacteria. **a**, **f** Plates and corresponding graphs showing disc-diffusion assay of the nanoparticles with increasing inhibition zones for *E. coli*. Inhibition zones obtained in similar assays with **b**, **g** *Agrobacterium tumefaciens*. **c**, **h** *Magnaporthe oryzae*. **d**, **i** MDR *E. coli*. **e**, **j** MDR *A. tumefaciens*. All experiments were done with increasing amounts of AuNPs on paper discs; clock-wise from top: 0.249 μg (20%), 0.498 μg (40%), 0.747 μg (60%), 0.996 μg (80%), and 1.245 μg (100%) of AuNPs. Data are means ± SE of three replicates. Different letters indicate statistically significant differences among the samples (*P* < 0.05, Tukey’s HSD test). Effect of AuNPs on the growth curve of **k** *E. coli*, **l** *A. tumefaciens*, **m** MDR *E. coli*, and **n** MDR *A. tumefaciens*. Asterisks indicate significant differences to control (Student’s *t* test, *P* < 0.05). **o** Microscopy of control *A. tumefaciens* cells. **p** *A. tumefaciens* showing loss of cellular integrity after treatment with the AuNPs

The AuNPs exhibited strong antimicrobial activities against human bacteria as well as plant pathogenic bacteria and fungus. The antimicrobial activity of the nanoparticles was assayed using paper discs with increasing amounts of AuNP, i.e., 0.249, 0.498, 0.747, 0.996, and 1.245 μg. Human bacteria *E. coli* (DH5α), the plant pathogenic bacteria *A. tumefaciens* (LBA4404) and the plant pathogenic fungus *M. oryzae* were used. The AuNPs were inhibitory to all these microorganisms even at the lowest concentrations, and the inhibition zones increased proportionately to the increase in particle concentration (Fig. 6). Figure 6a–c shows that the inhibition zones for *E. coli* (DH5α), *A. tumefaciens* (LBA4404) and *M. oryzae*, respectively. Figure 6f–h shows the graph of the inhibition zones of these three microbes as a function of amount of AuNPs used. The fungal extract alone did not have any inhibitory effect. The comparative trend of inhibition for the three microbes indicates a greater inhibitory effect on DH5α compared to that of LBA4404 and *M. oryzae* (Additional file 2: Fig. S1a).

### Assay of antimicrobial activity of AuNPs using multi-drug-resistant human and plant pathogenic bacteria

Multi-drug-resistant (MDR) DH5α and LBA4404 carrying plasmids with resistance genes were made. The MDR DH5α carrying pUC19 was resistant to 100 μg/ml Ampicillin and 35 μg/ml Chloramphenicol. LBA4404 transformed with pCAMBIA2301 was resistant to 25 μg/ml Rifampicin and 50 μg/ml Kanamycin. The AuNPs showed potent inhibitory activity against MDR DH5α and MDR LBA4404 (Fig. 6d, e). Figure 6i, j are the graphs showing the increase in inhibition with increase in concentration of the nanoparticles. The comparative trend of inhibition for the two MDR bacteria indicates greater inhibitory zones for *A. tumefaciens* compared to that of *E.coli* (Additional file 2: Fig. S1b).

### Bacterial growth assay over a time course in presence of AuNPs

The growth curve of DH5α treated with AuNPs was significantly different from the control set (Fig. 6k, m). In the control sets of DH5α and MDRDH5α, log phase of the growth curve started within 2 h of inoculation, whereas in AuNP-treated DH5α and MDR DH5α, no growth was observed up to 6 h after inoculation. The growth curves reached stationary phase after 12 h in both control and treated cells, but the growth was significantly reduced in the case of the treated bacteria. The LBA4404 growth curve showed initiation of log phase at 4 h after inoculation in the control set versus 6 h in the AuNP treated set. Similar effect on the growth curve of MDR LBA4404 was observed though in the control MDR LBA4404, the log phase started within 2 h (Fig. 6l, n).

### Effect of AuNPs on bacterial cell morphology and viability

In general, most nanoparticles can efficiently adhere onto cell membranes, get adsorbed, and thereafter affect the cell integrity [31]. Normal *A. tumefaciens* bacteria are rod shaped with clear outlines (Fig. 6o). The bacteria when incubated with the AuNPs showed distorted morphology, disintegration of the outer membrane resulting in irregular outline, and loss of integrity of cell shape and size (Fig. 6p). This is in accordance with that observed previously in *E. coli* cells treated with silica nanoparticles [32]. We found that incubation of bacterial cells with the nanoparticles for longer periods showed complete disintegration of cells.

### Fungal spore germination assay

**Fig. 7:**
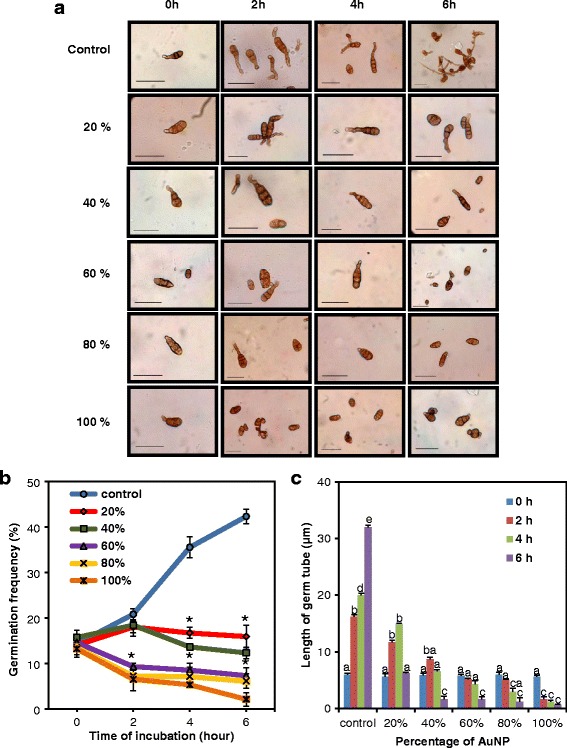
Effect of the AuNPs on the spore germination of plant pathogenic fungus *Alternaria solani*; **a** Spores after treatment with AuNPs (bar = 30 μm). Rows top to bottom: control spores followed by spores treated with different dilutions of AuNPs (15.56 mg/L stock). Columns left to right: increasing time of incubation with AuNPs showing least germination at 6 h incubation with 100% AuNPs. **b** Spore germination frequency (%) at different concentrations of AuNPs as a function of time of incubation. Asterisks indicate significant differences to control (Student’s *t* test, *P* < 0.05). **c** Average germ tube length at different concentrations of AuNPs as a function of time of incubation. Data represents means ± SE of three replicates. Different letters indicate statistically significant differences among the samples (*P* < 0.05, Duncan’s multiple range test). Spore germination frequency and the average germ tube length decreased with increasing doses of the AuNPs and increasing incubation period

The AuNPs were a potent suppressor of virulence of the plant pathogenic fungus *Alternaria solani*. Treatment of fungal conidia with increasing doses of AuNPs for different periods of incubation showed gradual decrease of germination frequencies and the lengths of the germ tubes (Fig. 7a). The representative pictures of conidia (Fig. 7a) and graphs show that the percentage of germination (Fig. 7b) and average lengths of the germ tubes (Fig. 7c) emerging from the fungal conidia decreased with increasing doses of the particles and increasing incubation periods. Thus, these AuNPs had significant antifungal property which is mediated through the suppression of germination of spores and retardation in the growth of hyphae.

Thus, these AuNPs have antimicrobial functions on bacteria as well as fungi, which is rarely reported for AuNPs. These AuNPs being toxic to multi-drug-resistant human bacteria can be utilized in treatment of MDR or extensively drug-resistant (XDR) bacteria-related human diseases which are difficult to treat. Furthermore, the antimicrobial effect against typical phytopathogens like *Agrobacterium* and fungi, it makes the particles suitable for as bacteriocides and fungicides in the form of nano-agrochemicals. These would enable sustainable management of crop loss through elimination of excessive and indiscriminate use of agrochemicals causing deterioration of soil health, degradation of agro-ecosystem, environmental pollution, and resistance in pathogens [33].

### The AuNPs have potent apoptogenic properties

**Fig. 8:**
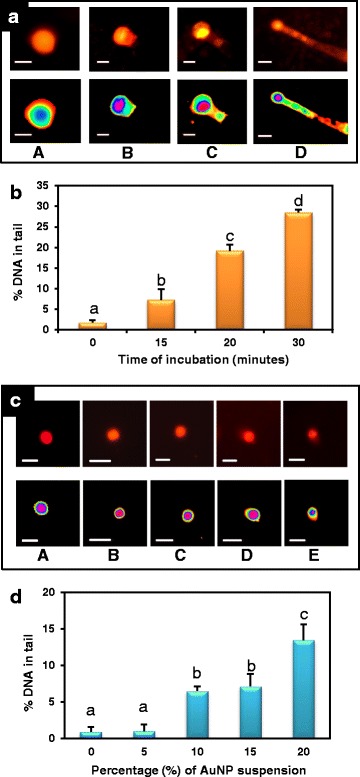
Assay of apoptotic properties of the AuNPs. **a** Comet assay and fluorescent microscopic images of nuclei of tobacco leaves treated with AuNPs for **A** 0 min. **B** 15 min, **C** 20 min, and **D** 30 min (bar = 5 μm). In the control sets (0 min incubation), most of the DNA is located in the head of the comet while cells subjected to longer treatment show increasing DNA damage and longer comet tails. **b** Mean of % tail DNA ± SE after different periods of incubation. Data represents means ± SE of three replicates. Different letters indicate statistically significant differences among the samples (*P* < 0.05, Tukey’s HSD test). **c** Assay of threshold dosage of AuNPs for apoptosis showing images of nuclei of tomato leaf cells treated with **A** 0% (control), **B** 5%, **C** 10%, **D** 15%, **E** 20% of AuNP suspension for 24 h (bar = 10 μm). **d** Mean % tail DNA ± SE after 24 h treatment with different concentration of AuNPs. Data represents means ± SE of three replicates. Different letters indicate statistically significant differences among the samples (*P* < 0.05, Tukey’s HSD test). Dosage below 10% show negligible DNA damage while higher than 20% show start of DNA damage

The single cell gel electrophoretic assay or comet assay is a sensitive method to compare apoptoic effects of materials [34, 35]. Treatment of tobacco leaf cells with 7.78 mg/L of AuNPs for a period of 15, 20, and 30 min resulted in gradual increase in apoptosis indicated by increase in percentage of DNA in tail (Fig. 8a, ‘A’, ‘B’, ‘C’, ‘D’). The maximum migration of DNA occurred after 30 min of treatment showing a tail DNA value 28.44 ± 0.74% which was significantly higher than that of untreated control cells (1.7 ± 0.59%). The incubation periods of 15 and 20 min caused less DNA migration with tail DNA values of 7.25 ± 2.56 and 19.19 ± 1.54%, respectively (Fig. 8b). Therefore, these AuNPs have the capacity to induce apoptosis in eukaryotic cells in higher doses. Recently, nanoparticles are being used in novel strategies to target and kill cancer cells. As illustrated by dynamic and quantitative imaging, successful application of nanoparticles as an alternative therapy for cancer depends on the apoptotic properties of the particles [36]. Hence, the present finding shows potent apoptogenic properties of these AuNPs which holds promise in future cancer therapy.

### These AuNPs are non-apoptogenic at lower doses

Safe application of metal nanoparticles as therapeutic agent needs a pre-determination of biological effect of the particles at the borderline toxicity [37]. A threshold level of nanoparticles required for apoptosis was obtained through treatment of tomato leaf cells with different concentration of AuNP for 24 h (Fig. 8c). Low concentration, e.g., 5% *v*/*v* of AuNP stock suspension (15.56 mg/L) did not show significant apoptogenic effect on tomato cells even after 24 h of exposure. The nuclei after 10% AuNP treatment showed 6.52 ± 0.63 percentage of DNA in tail which was higher than untreated control nuclei (0.95 ± 0.66 percentage of DNA in tail) and those treated with 5% AuNP suspension (1.04 ± 0.89 percentage of DNA in tail). Treatment of tomato leaf cells with 15 and 20% AuNP resulted in a slight rise in DNA damage showing 7.15 ± 1.70 and 13.47 ± 2.16 percentage of DNA in tail, showing significant apoptogenic effect (Fig. 8d). Thus, in lower doses apoptogenic effect is negligible holding the possibility for these nanoparticles to be used as antimicrobial agent or drug/gene delivery vehicle in eukaryotic cells.

### The AuNPs are protein-coated

**Fig. 9:**
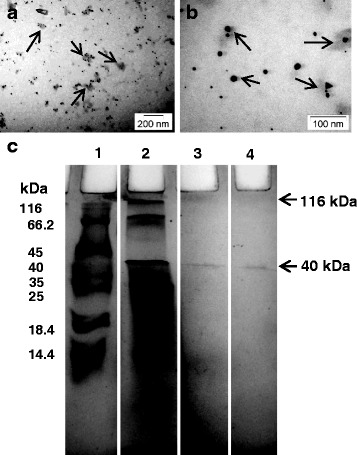
Protein cap analysis. **a**, **b** TEM images showing capping protein layer (arrows) around AuNPs. **c** SDS-PAGE of extracellular protein secreted from *T. crassum* and protein associated with nanoparticles. Lane 1, molecular size marker. Lane 2, total extracellular protein. Lane 3, nanoparticles loaded without boiling show faint protein band bound to the AuNPs at 116 kDa mark. There is also a band of detached protein at 40 kDa mark. Lane 4, nanoparticles after boiling with 1% SDS loading buffer showing disappearance of the 116 kDa band and a distinct 40 kDa band. Arrow indicates 40 kDa

A few previous studies have mentioned nanoparticles coated with protein of natural origin [15]. Lower magnification TEM showed that the AuNPs are surrounded by protein-like material (Fig. 9a, b). In order to confirm the nature of the material, the AuNPs were washed and run on SDS-PAGE along with cell-free extracts in other lanes (Fig. 9c). Boiling in SDS served to detach the surface bound proteins from the nanoparticles. The boiled nanoparticles (lane 4) showed the presence of a single intense band of 40 kDa which was similar to a protein band present in lane 2 (cell filtrate). However, in the sample that was not boiled (lane 3), a faint protein band bound to AuNP was seen at the 116 kDa level. Although another faint band did appear at the 40 kDa level due to dissociation of the capping proteins from the particles. Protein coats are known to promote stability of nanoparticles in solution and their catalytic activity [28, 38]. The naturally formed protein coat around the nanoparticles makes them functionally efficient for biomedical uses including easy adsorption and delivery of DNA or hydrophobic drugs [39]. Peptides and protein-aided delivery of AuNPs have been successfully used to overcome blood-brain barrier in treatment of central nervous system disorders [14]. Hence, these biocompatible AuNPs can be potentially suitable for several biomedical applications due to the small size, unique physico-chemical properties, and other advantages.

### The AuNPs could deliver green fluorescence protein (GFP) gene into Sarcoma 180 cancer cell lines

**Fig. 10:**
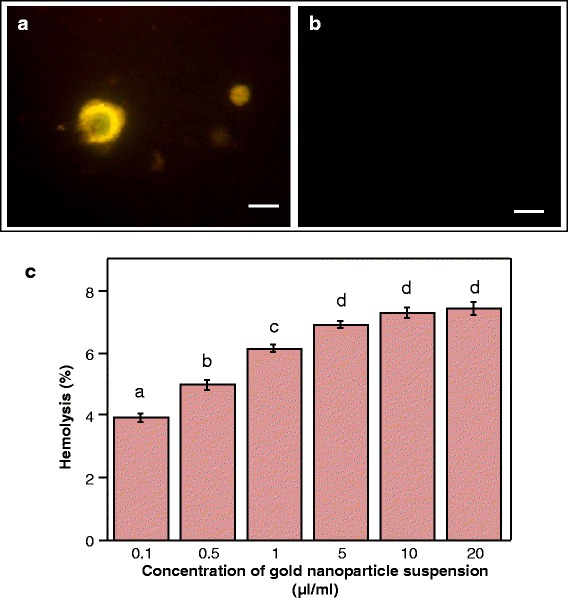
Gene delivery using AuNPs into Sarcoma 180 cancer cells and hemolysis assay with human erythrocytes; fluorescence microscopic image of **a** cancer cells expressing green fluorescence protein after uptake of DNA-AuNP complex and **b** control cells treated with free plasmid DNA (bars = 20 μm). **c** Percentage hemolysis with different dilutions of AuNPs. Data represents means ± SE of three replicates. Different letters indicate statistically significant differences among the samples (*P* < 0.05, Tukey’s HSD test)

Plasmid DNA pCAMBIA1302 harboring *gfp* marker gene, complexed with AuNPs, was used to treat Sarcoma 180 cells. The cells produced green fluorescence indicating uptake of plasmid DNA/AuNPs complex and subsequent expression of the gene in the cancer cell, while the cells treated only with naked plasmid DNA did not show the fluorescence (Fig. 10a, b). This confirms the high potential of these particles to not only deliver genes into cancer cells, the genes were stably expressed and remained functional once delivered into the cells.

Earlier metallic nanoparticles have been shown to exhibit immense therapeutic potential in treating variety of diseases like retinal neovascularization, HIV, Dalton’s lymphoma, and exhibited activity against hepatitis virus, respiratory syncytial virus, and herpes simplex virus [18]. Congruent to these reports, gold nanocarrier-based drug delivery in present study using AuNPs can be considered as a prospective mediator in numerous medical applications including diagnostics, drug delivery, and cancer therapeutics.

### Compatibility of the AuNPs with human erythrocytes and toxicity assay

Erythrocytes are simple and convenient model of the cell membrane system and used for studying nanoparticle-membrane interactions [40]. The hemolytic assay elucidates membrane-lytic activity of the AuNPs at different concentrations. Figure 10c shows that membrane-lytic activity of the nanoparticles was negligible at low concentrations. The highest hemolytic activity found at higher concentrations of nanoparticle suspension (up to 20 μl/mL) was less than 8% which indicates very low blood toxicity. The gradually increasing hemolytic activity with increasing concentration of AuNPs is likely due to increased affinity and adhesion of larger number of particles with the erythrocytes. This affinity of the particles to cell membranes is expected to facilitate their cellular transport. Low hemolytic activity along with effective cellular uptake render nanoparticles highly suitable for the development of safe and efficient theranostic agents [41].

## Conclusions

Use of edible mycorrhizal fungus to synthesize AuNPs of different geometric shapes in short reaction time with natural protein coat make this method simple and unique. The protein coat coming from the edible fungus did not have appreciable toxic effects and favor easy attachment of DNA onto the surface of the particles. Overall, these AuNPs show promise as antimicrobial, apoptotic agents for gene delivery into cancer cells.

Since filamentous fungi can withstand flow pressure or agitation [15], *T. crassum* can be cultured in fermentors to produce AuNPs on a large-scale using non-toxic agricultural wastes, allowing for easy withdrawal of product and system replenishing options [2]. Since the AuNPs are of different geometric shapes, there is the scope shape-based assortment with mechanical means such as centrifugation [42] and can be utilized according to their specific shape-based properties.

## Additional file

Additional file 1: **Table S1.** List of XRD peaks. (DOCX 12 kb)

Additional file 2: **Figure S1.** Graphs showing the comparative trend of inhibition of microbes in paper disc assays. (a) Greater inhibitory effect on *E. coli* compared to that of *A. tumefaciens* and the fungus *Magnaporthe oryzae.* (b) Greater inhibitory zones for multi-drug-resistant *A. tumefaciens* compared to that of multi-drug-resistant *E. coli*. (PPTX 71 kb)
