# Supplementary material for: Evaluating the antimicrobial, apoptotic, and cancer cell gene delivery properties of protein-capped gold nanoparticles synthesized from the edible mycorrhizal fungus Tricholoma crassum
Source: Nanoscale Res Lett. 2018 May 16;13:154. doi: 10.1186/s11671-018-2561-y (PMC5955874; doi:10.1186/s11671-018-2561-y)
Supplement: Supplementary file 1 — Table S1. List of XRD peaks. (DOCX 12 kb) [file 11671_2018_2561_MOESM1_ESM.docx]

**Supplementary Table 1**. List of XRD peaks

| Pos. [°2Th.] | d-spacing [Å] | Rel. Int. [%] Facets |
| --- | --- | --- |
| 38.2181 | 2.35496 | 100.00 111 |
| 44.3140 | 2.04414 | 27.77 200 |
| 64.6012 | 1.44273 | 15.13 220 |
| 77.5773 | 1.23065 | 16.10 311 |
| 81.6291 | 1.17852 | 3.82 222 |
