# Supplementary figures and images for: Evaluating the antimicrobial, apoptotic, and cancer cell gene delivery properties of protein-capped gold nanoparticles synthesized from the edible mycorrhizal fungus Tricholoma crassum
Source: Nanoscale Res Lett. 2018 May 16;13:154. doi: 10.1186/s11671-018-2561-y (PMC5955874; doi:10.1186/s11671-018-2561-y)

## Slide 1
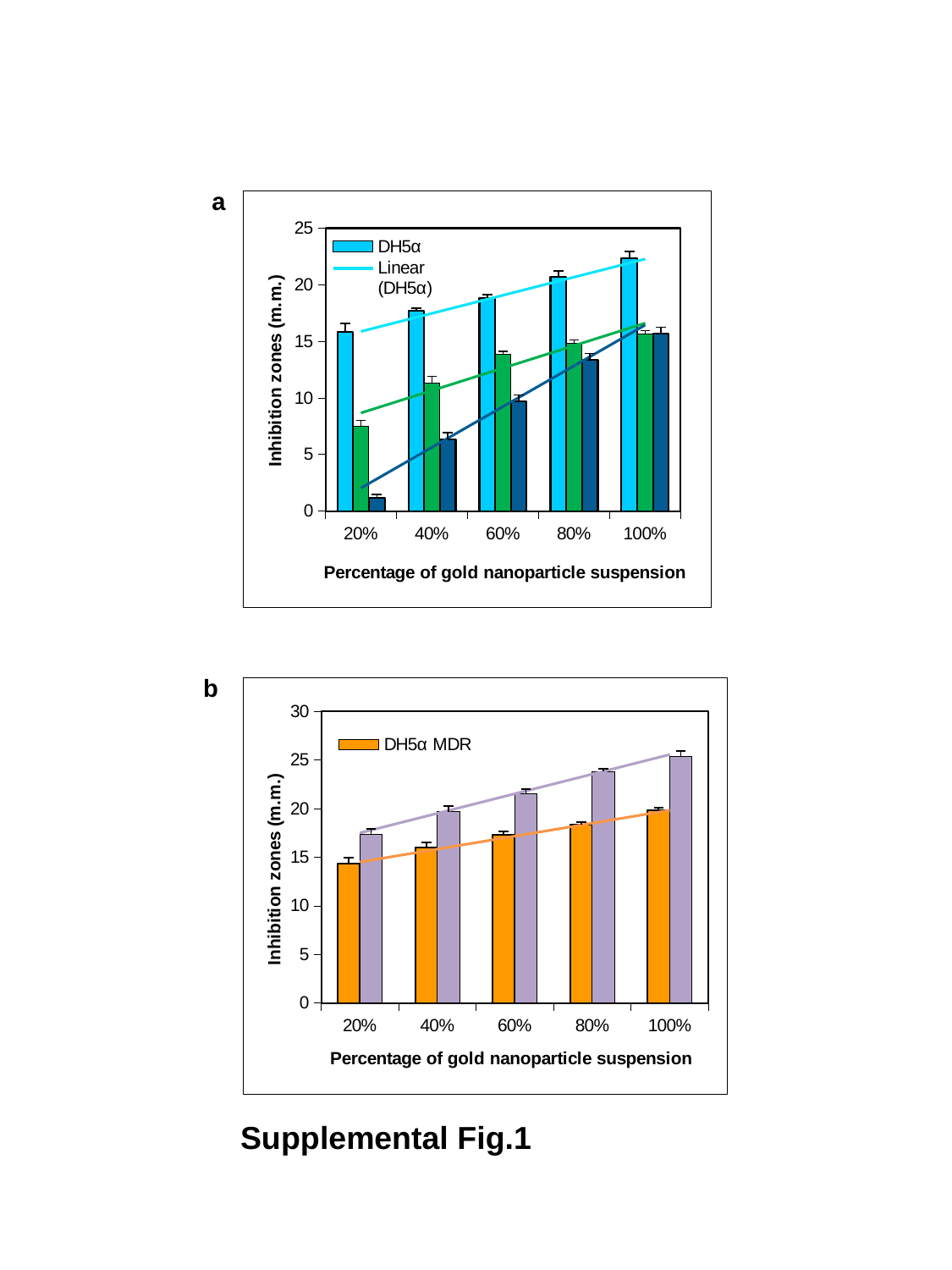

a
### Chart
| Category | DH5α | | M. oryzae |
|---|---|---|---|
| 0.2 | 15.833333333333334 | 7.5 | 1.1666666666666667 |
| 0.4 | 17.666666666666668 | 11.333333333333334 | 6.333333333333373 |
| 0.60000000000000064 | 18.83333333333311 | 13.833333333333334 | 9.666666666666677 |
| 0.8 | 20.666666666666668 | 14.833333333333334 | 13.333333333333334 |
| 1 | 22.33333333333311 | 15.666666666666726 | 15.666666666666726 |b
### Chart
| Category | DH5α MDR | LBA 4404 MDR |
|---|---|---|
| 0.2 | 14.333333333333334 | 17.33333333333311 |
| 0.4 | 16.0 | 19.666666666666668 |
| 0.60000000000000064 | 17.33333333333311 | 21.5 |
| 0.8 | 18.33333333333311 | 23.83333333333311 |
| 1 | 19.83333333333311 | 25.33333333333311 |Supplemental Fig.1

Supplement: Supplementary file 2 — Figure S1. Graphs showing the comparative trend of inhibition of microbes in paper disc assays. (a) Greater inhibitory effect on E. coli compared to that of A. tumefaciens and the fungus Magnaporthe oryzae. (b) Greater inhibitory zones for multi-drug-resistant A. tumefaciens compared to that of multi-drug-resistant E. coli. (PPTX 71 kb) [file 11671_2018_2561_MOESM2_ESM.pptx]
